# Genetic ablation of GINIP-expressing primary sensory neurons strongly impairs Formalin-evoked pain

**DOI:** 10.1038/srep43493

**Published:** 2017-02-27

**Authors:** Louise Urien, Stéphane Gaillard, Laure Lo Re, Pascale Malapert, Manon Bohic, Ana Reynders, Aziz Moqrich

**Affiliations:** 1 Aix-Marseille-Université, CNRS, Institut de Biologie du Développement de Marseille, UMR 7288, case 907, 13288 Marseille Cedex 09, France; 2 Phenotype Expertise, 5 Boulevard du Maréchal Koenig, 13009 Marseille, France

## Abstract

Primary sensory neurons are heterogeneous by myriad of molecular criteria. However, the functional significance of this remarkable heterogeneity is just emerging. We precedently described the GINIP^+^ neurons as a new subpopulation of non peptidergic C-fibers encompassing the free nerve ending cutaneous MRGPRD^+^ neurons and C-LTMRs. Using our recently generated *ginip* mouse model, we have been able to selectively ablate the GINIP^+^ neurons and assess their functional role in the somatosensation. We found that ablation of GINIP^+^ neurons affected neither the molecular contents nor the central projections of the spared neurons. GINIP-DTR mice exhibited impaired sensation to gentle mechanical stimuli applied to their hairy skin and had normal responses to noxious mechanical stimuli applied to their glabrous skin, under acute and injury-induced conditions. Importantly, loss of GINIP^+^ neurons significantly altered formalin-evoked first pain and drastically suppressed the second pain response. Given that MRGPRD^+^ neurons have been shown to be dispensable for formalin-evoked pain, our study suggest that C-LTMRs play a critical role in the modulation of formalin-evoked pain.

Deciphering the functional specialization of molecularly defined subpopulations of neurons is one of the most challenging issues in today’s neurobiology. Dorsal Root Ganglia (DRG) neurons represent a powerful model system to address this fundamental question. These neurons are highly heterogeneous by myriad of morphological, anatomical and molecular criteria. However, the functional significance of this remarkable diversity is under intense investigation within the sensory biology community. For example, genetic ablation of MRGPRD^+^ neurons led to a selective deficit in noxious mechanical pain sensitivity with no interference on noxious heat or cold sensation^1^. Pharmacological ablation of TRPV1 central projections selectively abolished noxious heat but not cold or mechanical sensitivity^1^. Interestingly, combined ablation of both subsets of neurons yielded an additive phenotype with no additional behavioral deficit^1^. In line with these findings, developmental ablation of Nav1.8-expressing neurons altered multiple sensory modalities, including an almost complete absence of the second phase of formalin-evoked pain, demonstrating, for the first time, that primary sensory neurons play an important role in sensing and transducing formalin-evoked pain^2^. Following this study, attempts to identify the specific subpopulation of neurons specialized in sensing and transducing formalin-evoked pain has been unsuccessful. Indeed, it has been shown that ablation of MRGPRD- and TRPV1-expressing neurons, both of which represent the vast majority of nociceptors, had no effect on formalin-evoked pain^3^, suggesting that formalin-evoked pain can be triggered by a small subset of neurons ablated in the Abrahamsen *et al*. study^2^. We and others have shown that low threshold mechanoreceptors Aβ, Aδ, C-LTMRs and the MRGPRB4^+^ neurons, express neither MRGPRD nor TRPV1 in mice^4^,^5^,^6^,^7^, implying that these populations of neurons are likely involved in sensing and transducing formalin-evoked pain. Here we used our recently engineered *ginip* versatile mouse model that allows an inducible and tissue specific ablation of GINIP-expressing neurons. We show that injection of diphtheria toxin selectively ablates MRGPRD^+^ neurons and C-LTMRs with no effect on Aβ and Aδ LTMRs or MRGPRB4^+^ neurons. Very interestingly, ablation of GINIP^+^ neurons significantly affected formalin-evoked first pain and strongly altered the second pain. As our genetic ablation approach selectively targets MRGPRD^+^ neurons and C-LTMRs, and knowing that MRGPRD^+^ neurons are dispensable for formalin-evoked pain, our results suggest that C-LTMRs play a critical role in formalin-evoked pain. Furthermore, in line with the selective ablation of C-LTMRs and the sparing of hairy skin innervating Aβ and Aδ LTMRs, GINIP-DTR mice displayed a partial but significant defect in the detection of touch-evoked sensation. Surprisingly, in contrast to MRGPRD-DTR mice, dual ablation of C-LTMRs and MRGPRD^+^ neurons had no effect on acute and injury-induced mechanical sensitivity, suggesting that C-LTMR and MRGPRD fibers may antagonize each other in sensing mechanical stimuli.

## Results

### Tissue specific and inducible ablation of GINIP-expressing neurons

**Figure 1:**
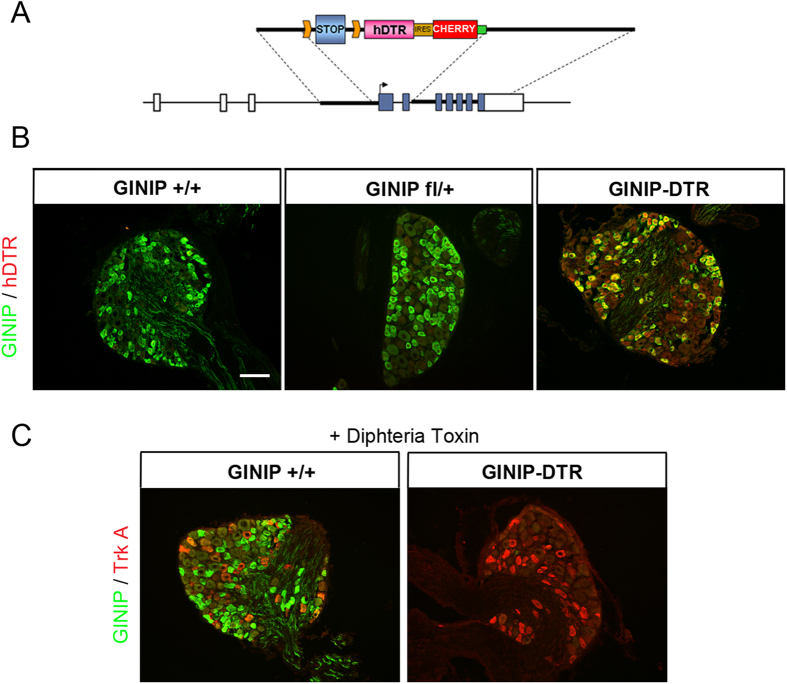
Selective ablation of GINIP^+^ neurons in adult DRGs. (**A**) Schematic representation of the construct used to target the *ginip* locus. GINIP-DTR mice were obtained by crossing GINIP^flx/+^ line with Nav1.8^cre/+^ mice. (**B**) Expression of hDTR is restricted to GINIP^+^ neurons. Double immunostaining using goat anti-hDTR (red) and rat anti-GINIP (green) antibodies on DRG sections from GINIP-DTR, GINIP^fl/+^ and GINIP^+/+^ littermates. hDTR expression is restricted to GINIP^+^ neurons, only in GINIP-DTR mice. Scale bar: 100 μm. (**C**) Injection of DT induced selective ablation of GINIP^+^ neurons only in GINIP-DTR mice. Double immunostaining using rabbit anti-TrkA (red) and rat anti-GINIP (green) antibodies shows a selective loss of GINIP^+^ in GINIP-DTR mice without affecting TrkA^+^ neurons.

In a recent study^6^, we generated a versatile mouse model that allows *ginip* gene global inactivation and an inducible and tissue-specific ablation of GINIP-expressing neurons (Fig. 1A). To gain insights into the *in vivo* functional specialization of GINIP-expressing neurons, we crossed GINIP^flx/+^ mice with mice expressing the CRE recombinase from Nav1.8 locus^2^,^8^. GINIP^flx/+^;Nav1.8^cre/+^ mice (hereafter GINIP-DTR mice) were undistinguishable from their WT littermates. Double labeling experiments using anti-GINIP and anti-hDTR antibodies showed the expression overlap between GINIP and hDTR only in GINIP-DTR but not in wild type (hereafter GINIP^+/+^ mice) or in GINIP^flx/+^ mice (Fig. 1B). This data demonstrates that CRE recombination occurs in a high fidelity manner and specifically targets neurons that drive expression of hDTR from *ginip* locus.

**Figure 2:**
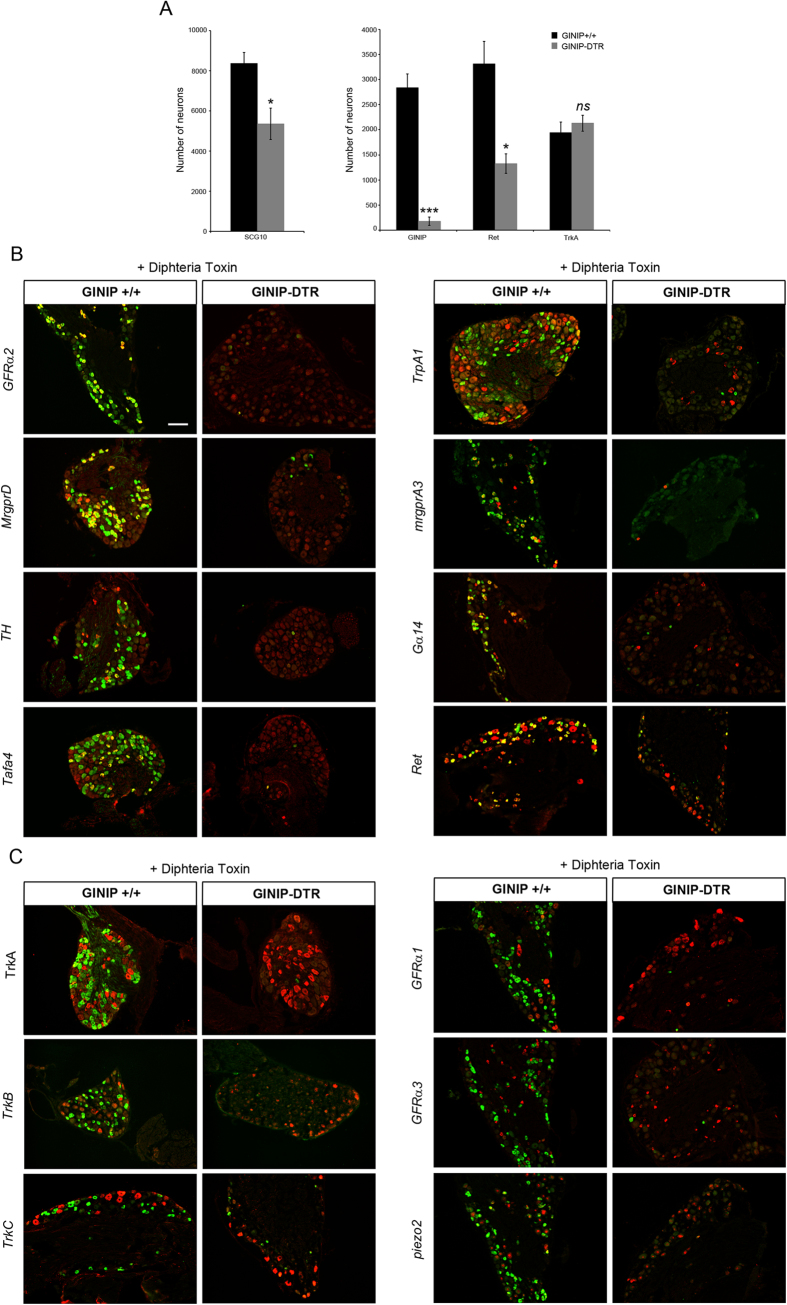
GINIP-expressing neurons ablation occurs in a cell specific manner. (**A**) Quantification of the total number of L4 DRGs neurons as well as the total number of neurons expressing the main markers of DRGs (n = 3 for each genotype). (***p < 0.001; *p < 0.05). (**B**) *In-situ* hybridization on DRG sections using antisense probes for genes that are known to be expressed in GINIP^+^ neurons (red). Each *in situ* hybridization is followed by immunostaining using rat anti-GINIP (green) to confirm the ablation of GINIP^+^ neurons in GINIP-DTR mice. Scale bar: 100 μm. (**C**) *In-situ* hybridization on DRG sections using antisense probes for genes that are known to be excluded from GINIP^+^ neurons (red). Each *in situ* hybridization is followed by immunostaining using rat anti-GINIP (green) to confirm the ablation of GINIP^+^ neurons in GINIP-DTR mice. Scale bar: 100 μm.

**Figure 3:**
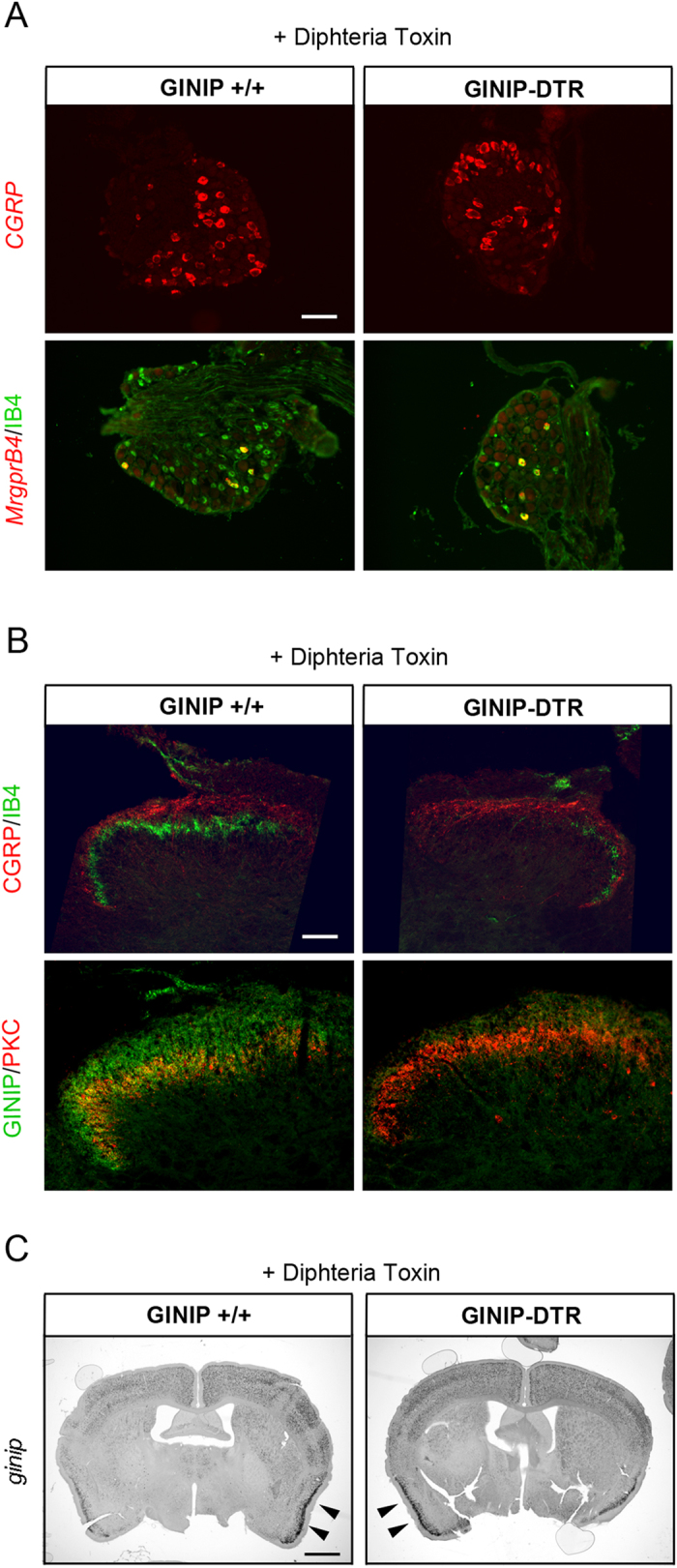
Ablation of GINIP^+^ neurons affects neither the central projections of the spared neurons nor the laminar distribution of PKCγ^+^ interneurons. (**A**) *In-situ* hybridization using *cgrp* and *mrgprB4* antisense probes and IB4 staining on DRG section from GINIP-DTR and GINIP^+/+^ injected with diphtheria toxin. Neuronal populations which do not express GINIP in the adult are not affected by DT injection in GINIP-DTR. Scale bar: 100 μm. (**B**) Double immunostaining using rat anti-GINIP, goat anti-CGRP and rabbit anti-PKCγ^+^ antibodies on spinal cord section from DT-injected GINIP-DTR and control mice. GINIP innervation (green) is completely gone in GINIP-DTR, whereas CGRP^+^ afferents (red) and PKC γ^+^ interneurons (red) are not affected. Note that IB4 residual neurons (green) in DRG are all *mrgprB4*^+^ and correspond to the residual IB4 staining in the lateral part of the spinal cord. Scale bar: 200 μm. (**C**) *In-situ* hybridization using *ginip* antisense probe on brain coronal sections from GINIP-DTR and GINIP^+/+^ injected with DT. GINIP^+^ neurons are not ablated in the brain. Strong staining in the cortex and in the piriform cortex (arrowhead) is still present in DT-injected GINIP-DTR mice. Scale bar: 1 mm.

Diphtheria toxin (DT) injection had no effect on GINIP^+^ neurons in GINIP^+/+^ mice and led to a selective and specific ablation of all GINIP^+^ neurons in GINIP-DTR mice without affecting the neighboring neurons expressing TrkA (Fig. 1C). To further characterize the selective ablation of GINIP-expressing neurons in GINIP-DTR mice, we performed a thorough quantitative and qualitative analysis of L4 DRGs using the pan-neuronal marker SCG10 in combination with a variety of DRG neuronal markers (Fig. 2A). Consistent with the previously described percentage of GINIP-expressing neurons in L4 ganglia, we found a 36% decrease in the total number of DRG neurons in GINIP-DTR mice (8367 ± 541 for the GINIP^+/+^ mice and 5360 ± 784 for GINIP-DTR mice, n = 3) (Fig. 2A). Accordingly, the total number of Ret+ neurons decreased by 60% in GINIP-DTR mice (3316 ± 446 for the GINIP^+/+^ mice and 1326 ± 192 for GINP-DTR mice, n = 3), whereas quantification of TrkA^+^ neurons showed no difference between GINIP-DTR and GINIP^+/+^ mice (Fig. 2A). Consistently, molecular markers that are expressed in GINIP^+^ neurons, such as *GFRα2, MrgprD, TH, Tafa4, TRPA1 low-expressors, mrgprA3, Gα14 and* the small diameter *Ret*^+^ neurons were massively or completely absent in DT-injected GINIP-DTR mice (Fig. 2B), whereas those that are excluded from GINIP^+^ neurons, such as TrkA, *TrkB, TrkC*, the subsets of *Ret*^+^ neurons expressing *GFRα1 and GFRα3, piezo2, CGRP* and *MrgprB4* were unaffected (Figs 2C and 3A). In line with these data, dorsal horn spinal projection of CGRP afferents, most of which express TrkA, occurs normally in DT-injected GINIP-DTR mice, whereas there was a massive decrease of IB4 afferents projections in laminae II of the dorsal horn spinal cord (Fig. 3B). Interestingly, IB4 afferents innervating the most lateral part of the spinal cord, known to express MRGPRB4^4^ are present in both animals (Fig. 3B). Finally, laminar organization of the dorsal horn appears normal as the PKCγ^+^ interneurons distribution remains intact in the GINIP-DTR mice (Fig. 3B). Very importantly, GINIP^+^ neurons in the brain were not affected by DT injection in GINIP-DTR mice as *ginip* transcripts are detected in GINIP^+/+^ as well as in GINIP-DTR brain slices (Fig. 3C). Altogether, these data show that our mouse model allows a highly controlled tissue specific and inducible neuronal ablation of GINIP^+^ neurons and suggest that the spared neurons undergo no changes both at the molecular and anatomical levels, thus opening the possibility to unravel the functional specialization of GINIP-expressing neurons in somatosensation in adult mice.

### GINIP-expressing neurons are dispensable for temperature sensation

**Figure 4:**
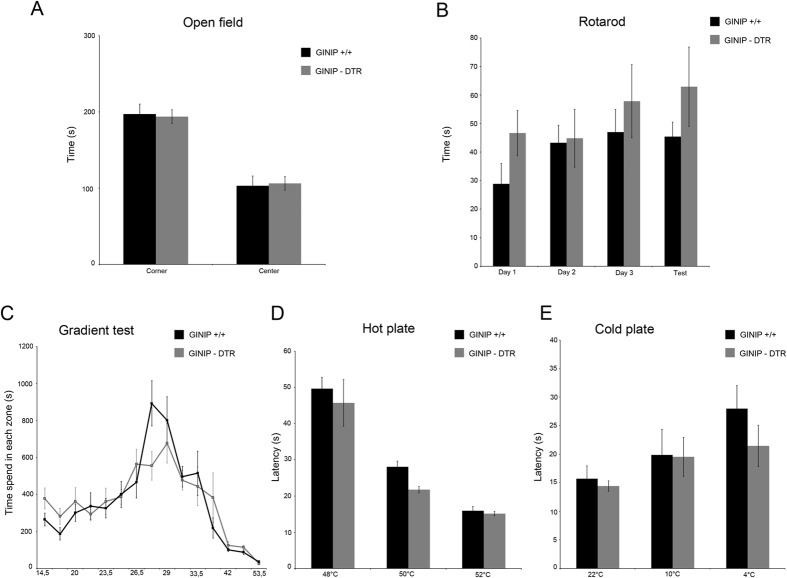
DT-injected GINIP-DTR mice display normal temperature and exploratory behavior. DT-injected GINIP-DTR and wild-type mice behave in the same way in the open field test (**A**) n = 8 and 10, respectively), the rotarod (**B**), n = 14 and 6, respectively), in the acute thermal gradient test (**C**), n = 10 and 9, respectively), in the hot plate test (**D**), n = 7 and 6, respectively) and in the cold plate test (**E**), n = 9 and 7, respectively).

To gain insights into the functional role of the GINIP^+^ neurons in somatosensation, we subjected GINIP-DTR mice to a large battery of somatosensory tests under acute and tissue or nerve injury conditions. GINIP-DTR mice have a normal body weight, and behave normally during the open field or rotarod tests, demonstrating that loss of GINIP^+^ neurons has no impact on motor activity or anxiety-like behaviors (Fig. 4A and B). We then subjected both genotypes to a variety of thermal tests including hot and cold plates and the thermal gradient tests. In these paradigms, GINIP-DTR mice behaved the same way as their GINIP^+/+^ littermates, suggesting that GINIP^+^ neurons are dispensable for the detection of temperature (Fig. 4C,D,E).

### Ablation of GINIP-expressing neurons causes a slight alteration of gentle touch sensation but not noxious or injury-induced mechanical sensitivity

**Figure 5:**
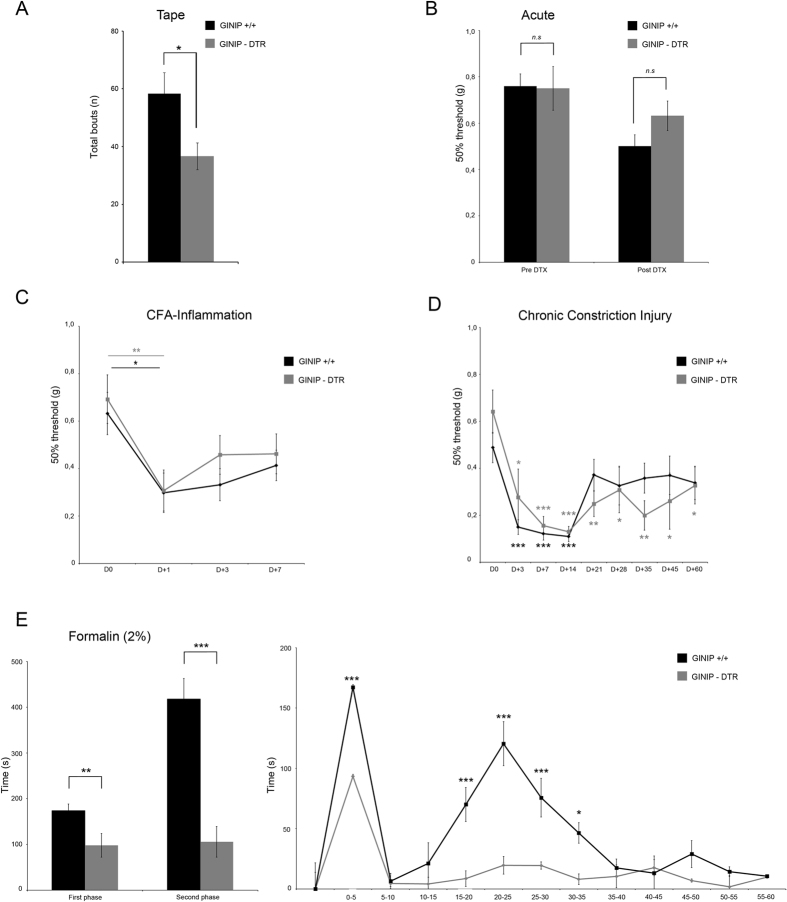
GINIP^+^ neurons are required for formalin-evoked pain hypersensitivity. (**A**) GINIP-DTR mice exhibited significantly less attempts to remove the tape from their back in comparison to the control mice (Fig. 5A, GINIP^+/+^ 58.3 ± 7.2 bouts n = 9 and GINIP-DTR 36.3 ± 4.6 bouts n = 11). (**B**) No difference in mechanical threshold between GINIP-DTR mice and GINIP^+/+^ littermate before (0.749 ± 0.09 and 0.759 ± 0.05/n = 10 and 10) or after DTX injection (0.631 ± 0.06 and 0.501 ± 0.05/n = 10 and 8, respectively). (**C**) No difference in CFA induced mechanical hypersensitivity between GINIP-DTR mice and GINIP^+/+^ littermate (n = 10 and 11, respectively). (**D**) GINIP-DTR and GINIP^+/+^ littermate developed a clear CCI-induced mechanical hypersensitivity during the first two weeks post injury with no significant difference (two-way RM ANOVA, t = 0,188, p = 0,854) between genotypes (n = 9 and 8, respectively). (**E**) Impaired formalin-evoked pain in DT-injected GINIP-DTR and GINIP^+/+^ littermate mice (n = 11 and 12 respectively). GINIP-DTR mice response to formalin-evoked pain is drastically altered with a moderate first phase (p = 0.006) and an almost complete abolition of the second phase (p < 0.001) compared to biphasic GINIP^+/+^ response.

We next tested the consequences of GINIP^+^ neurons ablation in mechanosensation. Given that C-LTMRs massively innervate the hairy part of the skin, we used the tape response assay to test how GINIP-DTR mice would react to a gentle mechanical stimulus applied to their hairy skin. In this assay, both genotypes had the same latency to the first response. However, GINIP-DTR mice exhibited significantly less attempts to remove the tape from their back in comparison to the control mice (Fig. 5A, GINIP^+/+^ 58.3 ± 7.2 bouts n = 9 and GINIP-DTR 36.3 ± 4.6 bouts n = 11). In a previous study, Cavanaugh and colleagues demonstrated that MRGPRD^+^ neurons play a critical role in acute and inflammation-induced mechanical pain^1^. Given that GINIP^+^ neurons encompass MRGPRD^+^ neurons and C-LTMRs^6^, we sought to analyze the mechanical sensitivity of GINIP-DTR mice under acute, inflammatory and nerve injury conditions using the Von Frey test. We found no differences in acute mechanical thresholds between GINIP^+/+^ and GINIP-DTR mice before and after DT injection (Fig. 5B). We also found that Completed Freund Adjuvant (CFA)- and Chronic Constriction nerve Injury (CCI)-induced mechanical sensitivity of GINIP-DTR mice was similar to that of their GINIP^+/+^ littermates (Fig. 5C and D). This data demonstrates that dual ablation of C-LTMRs and MRGPRD^+^ neurons does not recapitulate the acute and CFA-induced mechanical hyposensitivity due to the selective ablation of MRGPRD^+^ neurons alone, and suggests that C-LTMRs and MRGPRD^+^ neurons might play antagonistic roles in the modulation of acute and inflammation-induced mechanical sensitivity.

### GINIP-expressing neurons are required for formalin-evoked pain hypersensitivity

The formalin test is a widely used chemical test in pain research. However, the molecular mechanisms and the neuronal subpopulations underlying the nocifensive behavior triggered by formalin are largely unknown. Recent studies strongly suggested that a yet to be identified small subset of DRG neurons is required for formalin-evoked pain^2^,^3^. In GINIP-DTR mice, intraplantar injection of 10 μl of 2% formalin triggered a significant decrease in the formalin-evoked pain response during the first phase and a nearly complete absence of the second phase pain response (Fig. 5E). Of note, ablation of MRGPRD^+^ neurons alone or together with TRPV1^+^ neurons had no effect on formalin-evoked pain^3^. Combined ablation of both MRGPRD^+^ neurons and C-LTMRs led to a drastic deficit in formalin-evoked second phase pain hypersensitivity, indicating that GINIP-expressing neurons, most likely the C-LTMRs, are required for this pain process. This result is consistent with our previous finding in which we demonstrated that loss of TAFA4, a C-LTMRs- enriched chemokine-like protein, led to enhanced formalin-evoked pain specifically during the second phase^9^.

## Discussion

In this study, we used a genetic approach to selectively ablate the GINIP^+^ neurons encompassing two distinct subpopulations of cutaneous primary sensory neurons: MRGPRD^+^ neurons and C-LTMRs. We show that ablation of GINIP^+^ neurons affected neither the molecular contents nor the central projections of the spared neurons in GINIP-DTR mice, suggesting undetectable compensatory molecular or anatomical plasticity due to lack of GINIP-expressing neurons, and opening the possibility to unravel the functional specialization of GINIP^+^ neurons.

MRGPRD^+^ neurons have been described to play a critical role in mechanical pain^1^,^3^, whereas C-LTMRs ensure a dual function: they sense gentle touch under normal conditions^10^ and contribute to mechanical pain under pathological conditions^9^. GINIP-DTR mice had normal acute and inflammation-induced mechanical sensitivity, exhibited a slight abnormality in sensing gentle touch and nerve injury-induced mechanical pain and displayed a drastic alteration of formalin-evoked pain.

The formalin test is a valid, reliable and tonic model of continuous pain^11^. However, the neuronal subpopulations underlying the nocifensive behavior triggered by formalin are largely unknown. Genetic ablation of Nav1.8-expressing neurons completely abolished the second phase of formalin-evoked pain^2^, demonstrating that DRG neurons largely contribute to the prototypical biphasic pain response evoked by formalin injection. A follow up study from Shields and colleagues showed that MRGPRD^+^ and TRPV1^+^ neurons, both of which were largely eliminated in Nav1.8-DTA mice, were dispensable for formalin-evoked pain^3^, demonstrating that formalin-evoked nocifensive behavior requires a small population of primary sensory neurons. Here, we show that ablation of GINIP^+^ neurons led to a significant decrease in the first phase and a nearly complete abolition of the second phase of formalin-evoked pain. Given that GINIP is expressed in MRGPRD^+^ neurons and in C-LTMRs and that this protein is totally excluded from TRPV1^+^, MRGPRB4^+^, Aβ, and Aδ low threshold mechanoreceptors^6^, our results suggest that C-LTMRs likely represent the subpopulation of neurons that contribute to the modulation of formalin-evoked pain.

**Figure 6:**
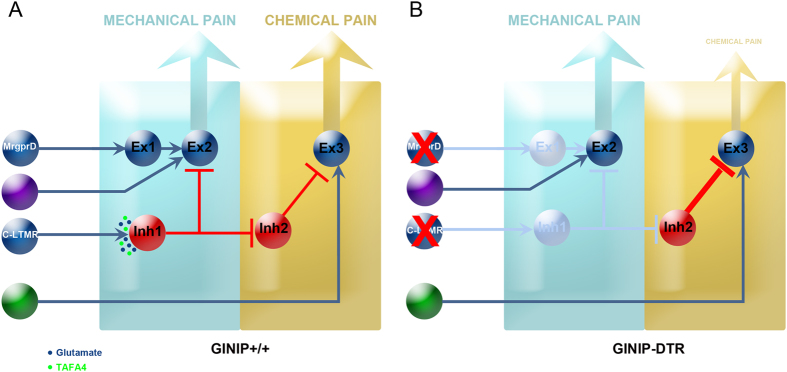
An integrated model. In GINIP^+/+^ mice (**A**), paw injection of formalin activate local sensory neurons (green neurons) that excite the excitatory interneuron 3 located in the superficial layers of the spinal cord. The intensity and duration of formalin-evoked pain is under the control of an inhibitory tone mediated by C-LTMRs. Licking, flinching and biting behavior activate C-LTMRs which release both glutamate and TAFA4. Glutamate activates inhibitory interneuron 1, freeing excitatory interneuron 3 from the inhibitory effect of inhibitory interneuron 2, thus ensuring normal transmission of formalin-evoked pain to projection neurons. TAFA4 co-released by C-LTMRs modulates the amount of pain thus avoiding excess of formalin-evoked pain. In line with this, TAFA4 null mice display exaggerated response to formalin during the second phase. Our model also provides a rational explanation of how C-LTMRs regulate noxious mechanical information flow from nociceptors as previously described by Lu and Perl. In GINIP-DTR mice (**B**), loss of C-LTMRs opens the gate for inhibitory interneuron 2 to exert a strong inhibitory tone on the excitatory interneuron 3, leading to abolition of formalin-evoked pain. Our model predicts that loss of C-LTMRs would exacerbate acute and injury-induced mechanical sensitivity. Cavanaugh *et al*. showed that loss of MRGPRD^+^ neurons led to acute and inflammation-induced mechanical hyposensitivity. In this study, mice lacking MRGPRD^+^ neurons and C-LTMRs exhibited normal mechanical sensitivity, suggesting that the hyposensitivity due to loss of MRGPRD^+^ neurons is counterbalanced by the hypersensitivity due to loss of C-LTMRs. A definite answer to this hypothesis will be provided by the selective ablation of C-LTMRs.

How a population of neurons known to exclusively innervate the hairy skin, could modulate pain that is evoked by an inflammatory agent injected in the glabrous skin? The most plausible explanation to this question resides on the type of response that formalin injection triggers in the mice. Indeed, upon formalin injection, mice will vigorously shake their paw; they will also grab it and intensely lick it from all sides, sometimes up to the tibial area. This behavior which consists of strong and repetitive innocuous mechanical stimuli applied to both glabrous and hairy skin of the hind paw will activate low threshold mechanosensory neurons, including C-LTMRs. The next question is how these shaking, licking and biting behaviors modulate formalin-evoked pain? The answer to this question is depicted in our working model shown in Fig. 6 which is largely inspired from a recent short review by Arcourt and Lechner^12^. In this model, we propose that C-LTMRs connect an inhibitory interneuron, connected to a second inhibitory interneuron, which itself is connected to an excitatory interneuron. With such model, in WT mice, mechanical activation of C-LTMRs will lead to the release of glutamate and TAFA4. Glutamate and TAFA4 will exert opposing actions: glutamate will activate the first inhibitory interneuron that will repress the inhibitory tone of the second inhibitory interneuron on the excitatory interneurons thus promoting formalin-evoked pain. On the other hand, TAFA4 will limit the glutamate-mediated activation of the first inhibitory interneuron to control the intensity of formalin-evoked pain. Accordingly, in the TAFA4 knock-out mice, the TAFA4 modulatory effect is no longer excreted leading to exacerbated formalin-evoked second pain^9^. In GINIP-DTR mice, loss of C-LTMRs will fail to activate the first inhibitory neuron, freeing the second inhibitory neuron to silence the excitatory interneuron, thus decreasing the first pain and preventing the onset of the second phase of formalin-evoked pain. Further investigations aimed at confirming/consolidating this putative working model are warranted.

Our behavioral studies also showed that GINIP-DTR mice exhibit a slightly impaired gentle touch sensation, further consolidating the role of C-LTMRs in light touch sensation. They also revealed that GINIP-DTR mice display normal acute and injury-induced mechanical sensitivity. Impressively, this later phenotype is opposite to that described by Cavanaugh and colleagues who showed that selective ablation of MRGPRD^+^ neurons caused strong mechanical hyposensitivity under acute and CFA-induced inflammation^1^, suggesting that C-LTMRs and MRGPRD^+^ neurons play antagonistic roles in modulating acute and injury-induced mechanical sensitivity. Support for this hypothesis can be found in different studies: Zhang and colleagues^13^ have shown that ablation of MRGPRD^+^ neurons reduced the firing of superficial dorsal horn nociceptive-specific neurons in response to graded mechanical stimulation, and Lu and Perl^14^ identified a neural circuitry in the substantia gelatinosa in which innocuous impulses activating C-LTMRs suppress nociceptive inputs. Based on our model we can postulate that under inflammatory and nerve injury conditions, C-LTMRs-derived TAFA4 becomes dominant over glutamate, thus reducing glutamate-mediated activation of the first inhibitory interneuron, leading to disinhibition of excitatory interneuron 2 and increased mechanical hypersensitivity. In the absence of MRGPRD^+^ neuron and C-LTMRs, the gate is open for the violet primary sensory neuron to activate excitatory interneuron 2 thus explaining the opposite phenotypes between MRGPRD-DTR and GINIP-DTR mice.

In conclusion, although we ablated two distinct subpopulations of neurons, our study strongly suggests that C-LTMRs is likely the subpopulation of neurons responsible for the modulation of formalin-evoked pain and consolidates a previous study suggesting that C-LTMRs negatively modulate inputs from nociceptors through an excitatory drive onto GABAergic interneurons in lamina II. Our study also encourages finding the best genetic approach to selectively eliminate C-LTMRs.

## Materials and Methods

### Mice

Mice were maintained under standard housing conditions (23 °C, 40% humidity, 12 h light cycles, and free access to food and water). GINIP^flx/+^ mice were previously generated in the laboratory^6^. Special efforts were made to minimize the number as well as the stress and suffering of mice used in this study. All protocols are in agreement with European Union and national recommendations for animal experimentation and have been approved by “le ministère de l’éducation nationale, de l’enseignement superieur et de la rechercherche” under the reference number: APAFIS#1537-2015070217242262v6

Diphtheria Toxin (20 μg/kg) was injected i.p. on 2 days; separated by 72 h. Behavioral tests were performed 2 to 4 weeks after the initial DT injection.

### *In situ* hybridization and immunofluorescence

*In situ* hybridization and immunofluorescence were carried out following standard protocols^15^. To obtain adult tissues, animals were deeply anesthetized with a mix of ketamine/xylazine and then transcardially perfused with an ice-cold solution of 4% paraformaldehyde in PBS. Then, DRGs and spinal cord were dissected; they were post-fixed ON in the same fixative at 4 °C. Tissues were then transferred into a 30% (w/v) sucrose solution for cryoprotection before being frozen 24 h later and stored at −80 °C. Samples were sectioned at 12 μm (DRG section) or 16 μm (spinal cord section) using a standard cryostat (Leica).

RNA probes were synthesized using gene-specific PCR primers and cDNA templates from mouse DRG. *In situ* hybridization was carried out using digoxigenin labeled probes. Probes were hybridized overnight at 55 °C and the slides incubated with the horseradish peroxidase anti-digoxigenin antibody (Roche). Final detection was achieved using cy3 TSA plus kit (Perkin Elmer). The following oligonucleotides were used for the nested PCRs for probe synthesis:

*GINIP-F1: CAGGATAGGTGGGACAGAGAAG*,

*GINIP-R1: ATGTATCTCCTGCCTGCTTCAT*,

*GINIP-F2: TACCTGCTATGGATC*,

*GINIP-R2* + *T7: TAATACGACTCACTATAGGGTTCTCCTGAAACCAT*,

*MrgprD-F1: GGGCATCAACTGGTTCTTACTC*,

*MrgprD-R1: AGGGATTGTCTTGACTGTCG*,

*MrgprD-F2: AACGGGATGTGAGGCTACTTTA*,

*MrgprB4-F1: GGACCTGTGCCAGATATTCC*,

*MrgprB4-R1: GGACCCCTCTCTCCACTCTC*,

*MrgprB4-F2: CAGGAATGCCAGTGGAAAAT*

*MrgprB4-R2* + *T7: TAATACGACTCACTATAGGGCATCGCAACCTGTGTTGTCT*,

*TrkB-F1: CTGAGAGGGCCAGTCACTTC*,

*TrkB-R1: CATGGCAGGTCAACAAGCTA*,

*TrkB-F2: CAGTGGGTCTCAGCACAGAA*,

*TrkB-R2* + *T7: TAATACGACTCACTATAGGGCTAGGACCAGGATGGCTCTG,*

*SCG10-F1: GCAATGGCCTACAAGGAAAA*,

*SCG10-R1: GGCAGGAAGCAGATTACGAG*,

*SCG10-F2: AGCAGTTGGCAGAGAAGAGG*,

*SCG10R2* + *T7: TAATACGACTCACTATAGGGGGCAGGAAGCAGATTACGAG*.

*Gfrα1-F1: TCTGTCCCCTGTCCTCTTGTAT*

*Gfrα1-R1: CGCAACCATTAACAAATTCTGA*

*Gfrα1-F2: GCTTCAGGGGACTGTTTGTAAC*

*Gfra1-R2* + *T7: TAATACGACTCACTATAGGGCCTCCTGCTCTGTGTACTTGTG*

*Gfra2-F1: CCTTTCTCCTCCCAAATTTCTT*

*Gfra2-R1: GCAACTCGCTTCCTAGTACGTT*

*Gfra2-F2: TCACTGGTGTTTTCTCTCTGGA*

*Gfra2-R2* + *T7: TAATACGACTCACTATAGGGACATTTTCGCTCATCTGTAGGG*

*Gfra3-F1: AGCAACCCTGCTCTGAGACT*

*Gfra3-R1: TTTAATCATGACCCAAGGGACT*

*Gfra3-F2: CTTTCTCCATCCTTCCCTTGAT*

*Gfra3-R2* + *T7: TAATACGACTCACTATAGGGGAATTTGGGGACAGTAATA*

*TH-F1: AAGCCAAAATCCACCACTTAGA*

*TH-R1: CCGTGGAGAGTTTTTCAATTTC*

*TH-R2* + *T7: TAATACGACTCACTATAGGGAGAGATGCAAGTCCAATGTCCT*

*Tafa4-F1: TGCTCAGAAGTTCATAGCCAAA*

*Tafa4-R1: TAAAGGAACATTTGCAAGCTCA*

*Tafa4-F2: ATATGTGCAGTGTGG*

*Tafa4-R2* + *T7: TAATACGACTCACTATAGGGCAGCCAAGTTCAAAC*

*TrpA1-F1: AATGGTGTGCCTATGGCTTC*

*TrpA1-R1: GGACCTCTGATCCACTTTGC*

*TrpA1-F2: ACCCATGACCCTTCTTGTTG*

*TrpA1-R2* + *T7: TAATACGACTCACTATAGGGCCACCTGCATAGCAATCCTC*

*MrgprA3-F1: GACCCTGATCCCAGACTTGA*

*MrgprA3-R1: CAGTGGAGAGCTTTGGAAGG*

*MrgprA3-F2: ATTGTGTTCTGGCTCCTTGG*

*MrgprA3-R2* + *T7: TAATACGACTCACTATAGGGACAGTGGTCAAGTGCAGCAG*

*TrkC-F1: CCCACACCTTTTACCACCAC*

*TrkC-R1: AAGCCACACTCAGGAGGAGA*

*TrkC-F2: CCTGCCAACGTCTTTAGAGC*

*TrkC-R2-T7: TAATACGACTCACTATAGGGATGATCCACCATCCACAGGT*

*Ret-F1: TTAGATCCCCTTTCCCTTTAGC*

*Ret-R1: GAGTGTCTGTGGCTACAACTGC*

*Ret-F2: CTGCTCATCACTAGCCACCA*

*Ret-R2* + *T7: TAATACGACTCACTATAGGGTGTGCCTTTCACACAAGCTC*

*Piezo2-F1: CGTGCAACACATAGCTCTTCTC*

*Piezo2-R1: CCCTACAGTACTTGTGGGAAGG*

*Piezo2-F2: CTGGTGGTTGGCAAGTTTGT*

*Piezo2-R2* + *T7: TAATACGACTCACTATAGGGGACGCAATGGGTAGGGACAC*

*Cgrp-F: TGCAGGACTATATGCAGATGAAA*

*Cgrp-R: GGATCTCTTCTGAGCAGTGACA*

For immunofluorescence, primary antibodies were diluted in PBS-10% donkey serum (Sigma), 3% bovine albumin (Sigma), 0.4% triton-X100 and incubated overnight at 4 °C. Primary antibodies used in this study are as follows: rabbit anti-TrkA 1:1000 (generous gift from Dr. L. Reichardt, University of California), goat anti-TrkC 1:500 (R&D systems), goat anti-Ret 1:500 and anti-hDTR 1:500 (R&D systems), goat anti-CGRP 1:1000 (Acris antibodies), rabbit anti-PKCγ 1:1000 (Santa Cruz Biotechnology), and rat anti-GINIP 1:2000 (our lab). Corresponding donkey or goat anti-rabbit, anti-rat and anti-goat Alexa 488, 555, or 647 (Invitrogen or Molecular probe antibodies) were used for secondary detection. Isolectin B4 conjugates with AlexaFluorR 488, 568 or 647 dye were used at 1:200 (Invitrogen). Acquisition of images was performed on AxioImager Z1 (Zeiss).

### Cell counts and statistical analysis

We adopted a strategy that has been previously validated for DRG cell counts^16^. Briefly, 12 μm serial sections of thoracic DRG were distributed on 6 slides which were subjected to different markers including the pan-neuronal marker *SCG10*. This approach allowed us to refer all counting’s to the total number of neurons (*SCG10*^+^). For each genotype, lumbar (L4) DRG were counted in three independent animals. All cell counts were conducted by an individual who was blind to mice genotypes. Statistical significance was set to p < 0.05 and assessed using one way ANOVA analysis followed by unpaired t-test.

### Behavioral assays

All behaviour analyses were conducted on littermate males 8–10 weeks old. Animals were acclimated for one hour to their testing environment prior to all experiments that are done at room temperature (~22 °C). Experimenters were blind to the genotype of the mice during testing. The number of tested animals is indicated in the figure legends section. Statistical significance was set to p < 0.05 and assessed using one way ANOVA analysis followed by unpaired t-test (for open-field and tape tests), two-way ANOVA followed by post-hoc Bonferroni t-test (for gradient assay), or two-way repeated measures ANOVA followed by post-hoc Bonferroni t-test (for rotarod, hot and cold test, formalin test, and CFA and CCI model pain) using SigmaPlot 12.5 software. All error bars represent standard error of the mean (SEM). Gradient, Thermal plates, open-field, and Von Frey apparatus were from BioSeb instruments France.

#### Open-field test

The Open-field test is commonly used to assess locomotor, exploratory and anxiety-like behavior. It consists of an empty and bright square arena (40 × 40 × 35 cm), surrounded by walls to prevent animal from escaping. The animals were individually placed in the center of the arena and their behavior recorded with a video camera over a 5 min period and the time spent in the corner versus the center of the arena is recorded.

#### Rotarod test

A rotarod apparatus (LSI Letica Scientific Instruments) was used to explore coordinated locomotor and balance function in mice. Mice were placed on a rod that slowly accelerated from 4 rpm to 44 rpm over 5 min and the latency to fall off during this period was recorded. The test was done 4 consecutive days. Each day, the animals were tested three times separated by at least 5 min resting period.

#### Temperature gradient assay

Response to the temperature gradient assay was performed as described previously^17^. Briefly, mice were individually video tracked for 90 min in four separate arenas of the thermal gradient apparatus (Bioseb). A controlled and stable temperature gradient of 14 °C to 55 °C was maintained using two Peltier heating/cooling devices positioned at each end of the aluminium floor. Each arena was virtually divided into 15 zones of equal size (8 cm) with a distinct and stable temperature. Floor temperature was measured with an infrared thermometer (Bioseb). The tracking was performed using a video camera controlled by the software provided by the manufacturer.

#### Hot plate test

To assess heat sensitivity, mice were placed individually on a metal surface maintained at 48°, 50°or 52 °C and the latency to nociceptive responses are measured (licking, shaking of hind paws or jumping). To prevent tissue damage, mice were removed from the plate immediately after a nociceptive response or a cut-off 90 s, 60 s and 45 s was applied respectively. Each mouse has been tested three times with a 5 min interval between each test. The withdrawal time corresponds to the mean of the three measures.

#### Cold plate test

To test cold sensitivity, mice were placed individually on a metal surface maintained at 22°, 10°, 4 °C or 0 °C. The rearing time of the mice is monitored for one minute. Each mouse is exposed three times to each temperature with a minimum of 5 min resting period between trials and one hour separating periods between temperatures.

#### Tape Response Assay

This test was achieved as described in Ranade and colleagues^18^. Briefly, a piece of 3 cm of tape was gently applied to the back of the mouse. Mice were then observed for 5 minutes and the total number of responses to the tape was counted. A response was scored when the mouse stopped moving and bit or scratched the piece of tape or showed a visible “wet dog shake” motion in an attempt to remove the foreign object on its back.

#### Formalin test

Mice were housed individually into Plexiglass chambers 20 min before injection. Following intraplantar injection of 10 μl of a 2% formalin solution (Fischer Scientific) into the left hind paw, time spent shaking, licking or lifting the injected paw was monitored for 60 min and analysed at 5 min intervals.

#### Von Frey test of mechanical threshold

Mice were placed in plastic chambers on a wire mesh grid and stimulated with von Frey filaments (Bioseb) using the up-down method^19^ starting with 1 g and ending with 2.0 g filament as cutoff value. Baseline measures of untreated or DT-treated WT or GINIP-DTR mice were performed on separate lots.

#### Complete Freund’s Adjuvant (CFA)-induced mechanical allodynia

We made an intraplantar injection of 10 μl of a 1:1 saline/CFA (Sigma, St. Louis, MO, USA) emulsion with a 30 gauge needle and measured mechanical thresholds one, three and seven days after the injection using the Von Frey hair filaments using the up-down method.

#### Unilateral peripheral mononeuropathy

For the chronic constriction of the sciatic nerve (CCI) model, unilateral peripheral mononeuropathy was induced in mice anaesthetized with Ketamine (40 mg/kg ip) and Xylasine (5 mg/kg ip) with three chromic gut (4_0) ligatures tied loosely (with about 1 mm spacing) around the common sciatic nerve^20^. The nerve was constricted to a barely discernable degree, so that circulation through the epineural vasculature was not interrupted^21^. For the chronic constriction model, mechanical allodynia was assessed before the surgery and three and seven days and then once a week post-surgery using the up-down Von Frey hair filaments method.

## Additional Information

**How to cite this article:** Urien, L. *et al*. Genetic ablation of GINIP-expressing primary sensory neurons strongly impairs Formalin-evoked pain. *Sci. Rep.* **7**, 43493; doi: 10.1038/srep43493 (2017).

**Publisher's note:** Springer Nature remains neutral with regard to jurisdictional claims in published maps and institutional affiliations.
